# Exploring Antibiotic-Mediated Disruption of Enterohepatic Circulation and Combined Oral Contraceptive Efficacy: A Systematic Review

**DOI:** 10.1089/whr.2024.0199

**Published:** 2025-05-19

**Authors:** David Elkhoury, Nithin Reddy, Deepti Venkatraman, Pruthvi Patel, Michael Montalbano

**Affiliations:** Anatomical Sciences, St. George’s University School of Medicine, Saint George’s, Grenada.

**Keywords:** oral contraceptives, antibiotics, contraceptive failure, pregnancy

## Abstract

**Background:**

Combined oral contraceptives (COCs) are essential for the well-being and reproductive health of millions of women globally. Despite their widespread use, concerns among clinicians persist about potential drug–drug interactions between antibiotics and COCs. This systematic review evaluates existing literature on the interaction between antibiotics and COC efficacy, offering guidance for clinicians in managing the use of contraception alongside antibiotics.

**Materials and Methods:**

Utilizing Preferred Reporting Items for Systematic Review and Meta-Analysis guidelines, a comprehensive literature search was conducted using PubMed, Clinical Trials.gov, Cochrane Library, and Google Scholar, focusing on studies published from 2000 to 2024. The search strategy was centered on peer-reviewed observational and experimental studies.

**Results:**

Initial analysis of the databases resulted in 712 potential articles. Nine articles were chosen relative to specific inclusion and exclusion criteria. While most antibiotics did not compromise COC effectiveness, enzyme-inducing antibiotics, particularly rifampicin, significantly reduced COC efficacy.

**Conclusion:**

Although common antibiotics pose minimal risk to COC effectiveness, clinicians should remain vigilant when prescribing enzyme-inducing antibiotics such as rifampicin. When these antibiotics are used, it is advisable to consider additional or alternative contraceptive methods to ensure continued pregnancy prevention.

## Introduction

A long-standing topic of discussion and research in the field of reproductive health is the effectiveness of oral contraceptive pills (OCPs). OCPs are thought to be used by 151 million women globally.^1^ OCPs are used not only to prevent unintended pregnancies but may also be an option to manage conditions such as menstrual disorders, dermatological issues, fibroids, migraines, and endometriosis pain.^2,3^

OCPs can be divided into three main categories: progestin-only pills (POPs), combined oral contraceptives (COCs) with estrogen and progestin, and extended-use pills with continuous hormones to prevent ovulation and reduce periods.^4^ Progestins such as norethindrone, drospirenone, and gestodene thicken cervical fluid, modify the lining of the uterus, and prevent ovulation. The presence of estrogen in oral contraceptives, usually in the form of ethinyl estradiol or mestranol, inhibits ovulation by enhancing progestin’s effect and suppresses ovulation by reducing follicle-stimulating hormone release, preventing follicular development.^4^ COCs are the most widely used, with over 100 million current users globally, resulting in most medical literature focusing on this form of contraception.^5^ However, POPs also provide a contraceptive choice for women with contraindications that prevent the usage of contraceptives containing estrogen.^6^

In all of the above categories, ensuring sufficient quantities of active hormones is crucial for getting the intended contraceptive effect.^7^ However, some medical professionals advise that there may be consequential drug–drug interactions between hormonal medications, including COCs and antibiotics. These concerns were initially raised in 1973 when a woman became pregnant while contemporaneously using both hormonal contraception and chloramphenicol.^8^ Beyond chloramphenicol, concerns about possible drug–drug interactions for tetracyclines, ampicillin, cotrimoxazole, and other broad-spectrum antibiotics have been raised.^9^

## Underlying Mechanisms: Enterohepatic Circulation and Estrogen Metabolism

There are two primary mechanisms by which antibiotics are thought to reduce the efficacy of oral contraceptives. First, enzyme-inducing medications such as rifampin and enzyme inhibitors such as ketoconazole and erythromycin can alter the metabolism of OCPs. The induction of hepatic microsomal enzymes from the cytochrome P450 (CYP) family can play a crucial role in how estrogen is metabolized in the liver, reducing plasma concentrations and increasing the risk of contraceptive failure and unintended pregnancies. Research has demonstrated that the use of medications that induce CYP enzymes can decrease the availability of estrogens and progestins such as levonorgestrel (LNG), etonogestrel (ENG), and desogestrel.^10^

Second, antibiotics may interfere with the intestinal and hepatic circulation of ethinyl estradiol.^11^ In a typical enterohepatic circulation, the liver conjugates ethinyl estradiol for release into the bile; intestinal microbiota then deconjugate these metabolites, allowing reabsorption into the portal circulation.^12^ In order to maintain estrogen at therapeutic levels in the body, enterohepatic circulation ensures that the hormone is recycled consistently and effectively.^13^ However, antibiotics can disrupt the enterohepatic cycling of estrogen by reducing bacteria in the small intestine responsible for hydrolyzing the glucuronide moiety of estrogen metabolites. This disruption consequently interferes with the active metabolism and reabsorption of estrogen, potentially leading to alterations in estrogen levels within the body.^11^ Through this mechanism, many antibiotics, including penicillins, tetracyclines, cephalosporins, macrolides, sulfonamides, metronidazole, and antituberculosis drugs such as rifampicin, have been implicated in reducing the effectiveness of COCs.^14^

There are diverging viewpoints regarding the potential for drug interactions between antibiotics and the efficacy of COCs. Controlled studies conclude that common non-enzyme-inducing antibiotics do not impair the effectiveness of oral contraceptives.^15^ However, observational studies published show a substantial sevenfold increase in unintended pregnancies among individuals taking antibiotics compared with controls.^16^ Between 2000 and 2020, the number of women using modern contraceptive methods, such as oral contraceptives, intrauterine devices, injectables, implants, and sterilization, grew by 188 million worldwide, underscoring the increasing reliance on contraceptives globally.^17^ The conflicting findings from these studies have posed a challenge for the medical community. Given this uncertainty, this study aims to provide an updated and comprehensive literature review for medical professionals and their patients.

## Methods

**FIG. 1.**
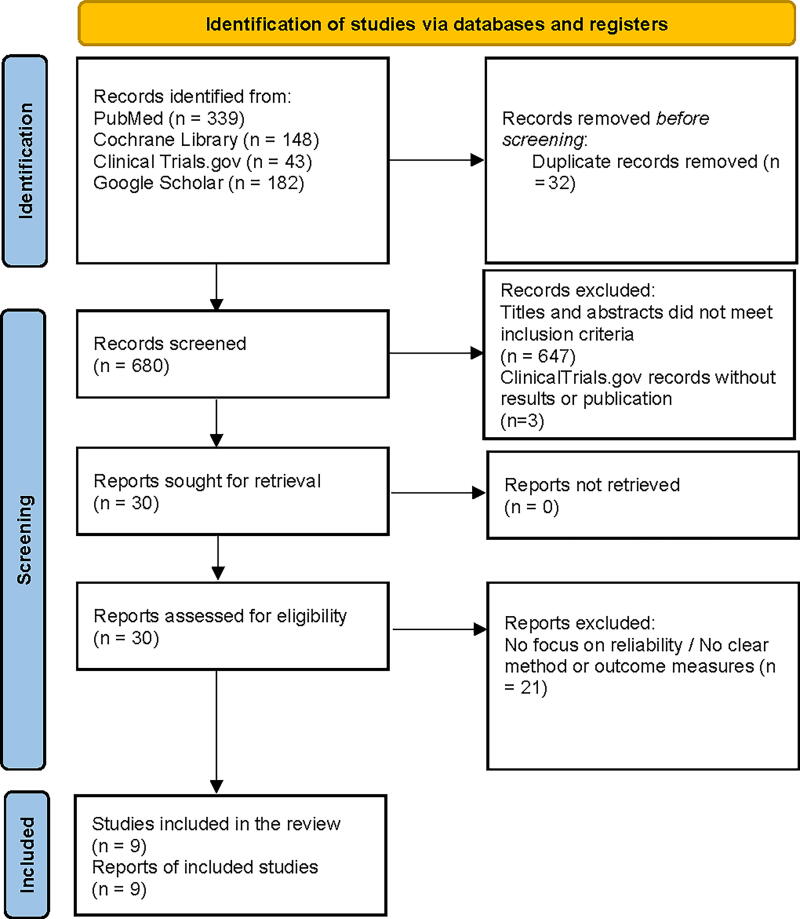
PRISMA flow diagram for systematic review based on inclusion and exclusion criteria. PRISMA, Preferred Reporting Items for Systematic Review and Meta-Analysis.

This systematic review adhered to the Preferred Reporting Items for Systematic Review and Meta-Analysis guidelines. A comprehensive literature search of PubMed, Clinical Trials.gov, Cochrane Library, and Google Scholar was conducted to identify articles published from January 1, 2000, to April 2, 2024. The search strategy centered on peer-reviewed observational and experimental studies, with detailed keywords and combinations provided in Supplementary Table S1. Duplicate publications, continued work of previous publications, and articles not available in English were excluded from the screening process. Additionally, opinion pieces, abstracts without full text, and editorial articles were excluded. Studies were excluded if they did not have a high-quality methodology defined as well-designed clinical trials, controlled drug interaction studies, validated models, or observational research that directly examined the effect of antibiotics on contraceptive efficacy. Studies were also excluded if they lacked clear methods or measurable outcomes or did not address antibiotic–contraceptive interactions. The screening evaluation was based on the abstract and then full text by four independent coauthors relative to the scope of this article according to the following criteria: peer-reviewed articles, full text, and focused on the direct interaction between antibiotics and oral contraceptives. Figure 1 presents the screening process for the literature review based on the inclusion and exclusion criteria.

## Results

A total of 712 articles were identified. Of these, 339 were found *via* PubMed, 148 through Cochrane Library, and 225 from other sources. After checking the titles and abstracts against the selection criteria, 30 potentially relevant studies were sought for full-text retrieval. Ultimately, six clinical trials and three observational studies were chosen. Information from the literature review was compiled and summarized in Table 1.

**Table 1.** Overview of Articles Selected and Reviewed

Author(s)	Country	Study design	Investigatory process	Findings
Clinical efficacy: contraceptive failure and unintended pregnancy				
Pottegård et al., 2018^18^	Denmark	Case–control study	Evaluation of Danish women who became pregnant between 1997 and 2015 while using OCP	No evidence for an elevated risk of OCP failure when using DCX
Koopmans et al., 2012^19^	Netherlands	Case–control study	Comparison of antibiotic exposure with self-matched controls in OCP failure pregnancies	No statistically significant change between exposed and controlled groups
Toh et al., 2011^20^	United States	Case–control study	Evaluated odds of antibiotic exposure during conception compared with control groups in OCP failure	No statistically significant change between the exposed and control groups
Jick et al., 2009^21^	United Kingdom	Nested case–control study	Evaluated the odds of unintended pregnancy while on OCPs in antibiotic users versus nonusers within 16 weeks of conception	No statistically significant difference between the exposed and controlled groups
Pharmacokinetic interactions: Hormonal metabolism and drug absorption				
Blode et al., 2012^22^	United States	Open-label study	Evaluated the effects of CYP3A4 induction using RIF and CYP3A4 inhibition using KTZ/EM to assess OCP pharmacokinetics	Systemic drug exposure was significantly reduced by concomitant RIF use, while systemic drug exposure was increased during coadministration of KTZ and EM
Pohl et al., 2013^23^	France	Single sequence crossover study	Assessed the effects of steady-state EM on the pharmacokinetics of ulipristal acetate by administering oral UPA	Coadministration of UPA with EM increased UPA’s absorption and metabolism due to CYP3A4 inhibition
Weisinger et al., 2020^24^	Germany	Randomized clinical drug–drug interaction study	Subjects were randomized into treatment groups and received different doses of RIF to induce CYP3A	Moderate CYP3A induction had limited effects, but robust induction significantly affected progestin exposure
Sunaga et al., 2021^25^	United States	Comparative analysis	Comparison of CYP3A4-inducing drugs on LNG and ENG/DSG	LNG and ENG are susceptible to adverse drug interactions with CYP3A4-inducing drugs
Radtke et al., 2023^26^	United States	Secondary analysis utilizing pharmacokinetic modeling	Analyzed various RIF dosing regimens on OCP clearance	Daily RIF usage may increase OCP clearance, while single doses or intermittent dosing every 4 weeks are unlikely to interact significantly with OCPs

DCX, dicloxacillin; DSG, desogestrel; EM, erythromycin; ENG, etonogestrel; KTZ, ketoconazole; LNG, levonorgestrel; OCP, oral contraceptive pill; RIF, rifampicin; UPA, ulipristal acetate.

## Discussion

### Contraceptive efficacy and drug interactions reviewed

This systematic review evaluates existing literature on the interaction between antibiotics and COC efficacy, offering guidance for clinicians in managing the use of contraception alongside antibiotics. There were no significant increases in contraceptive failures associated with antibiotic use. This analysis aligns with previously published data that most antibiotics do not interfere with the effectiveness of COCs.^18,20^ Likewise, there were no noticeable differences in the pregnancy rates of antibiotic users and nonusers, which corroborates the existing belief that antibiotics do not impact the effectiveness of contraceptives.^21^ This discrepancy in outcomes among different studies illustrates the multifaceted nature of the issue, which is influenced by local prescribing practices as well as patient adherence.^19^

Results indicated that rifampicin reduces the systemic exposure of contraceptives to the person by inducing CYP3A4. These findings further support earlier studies that underline the importance of enzyme modulation.^22^ On the contrary, erythromycin and other CYP3A4 inhibitors were found to increase the systemic exposure of ulipristal acetate, thereby demonstrating their important role in the metabolism of hormones.^23^

A comprehensive analysis of the data revealed variations concerning the contraceptive type and frequency of rifampicin use. Our findings corroborate those of the studies that stated that continuous rifampicin usage leads to an increased clearance of OCPs, whereas intermittent dosing has minimal interaction.^24,26^ Likewise, observational studies have noted that ENG and LNG-containing oral contraceptives have a predisposition to negative interactions with CYP3A4-inducing medications.^25^

### Historical context and broader literature review

Concerns regarding the effectiveness of oral contraceptives in conjunction with antibiotics have existed since the 1970s, owing to the belief that rifampicin reduces estrogen levels through hepatic enzyme induction.^27^ Research has similarly shown that while dirithromycin results in a modest reduction in ethinyl estradiol levels, ovulatory cycles are not observed, reinforcing the assertion that macrolide antibiotics do not significantly affect the efficacy of oral contraceptives.^28^ Similarly, another study revealed that ciprofloxacin does not significantly alter plasma ethinyl estradiol levels with the concurrent use of ampicillin.

Recent research suggests that some antibiotics, such as macrolide and ciprofloxacin, have little or no impact on the actions of ampicillin and its use as a contraceptive ampicillin.^28–30^ Similarly, a retrospective study across three dermatology practices found no increased pregnancy risk among oral contraceptive users taking antibiotics.^31^ In conjunction with others, this study adds to the body of evidence that most antibiotics assumed to limit contraceptive effectiveness do not have a significant impact.

## Summary of Global Guidelines

According to the World Health Organization (WHO) guidelines, most broad-spectrum antibiotics, antifungals, and antiparasitic agents have no significant pharmacokinetic effects with COCs.^32^ Also, the Faculty of Sexual and Reproductive Healthcare (FSRH), an entity within the Royal College of Obstetricians and Gynecologists, released guidelines in 2019 focusing on using combined hormonal contraception (CHC). These guidelines emphasize informing women using enzyme-inducing drugs that the effectiveness of CHCs can be reduced for 28 days after cessation of enzyme-inducing drugs. However, when non-enzyme-inducing antibiotics are taken for short periods in conjunction with COCs, no additional contraceptive measures are required.^33^ In addition, the Centers for Disease Control and Prevention (CDC) U.S. Medical Eligibility Criteria (USMEC) guidelines, endorsed by the American College of Obstetricians and Gynecologists (ACOG), state that broad-spectrum antibiotics pose minimal risk of interaction with OCPs.^34^ However, an exception is rifampin/rifampicin, which significantly increases the risk of reduced contraceptive efficacy. The CDC MEC is updated frequently, with the latest recommendations available in 2024.^35^

Women using rifampin are recommended to consider alternate contraceptive methods such as the LNG-releasing intrauterine device or depot medroxyprogesterone acetate, as these methods are not affected by enzyme-inducing drugs.^32^ ACOG, WHO, and FSRH guidelines confirm that broad-spectrum antibiotics, excluding enzyme inducers such as rifampin, do not affect the efficacy of hormonal contraceptives. Health care providers should continue to reassure patients that additional contraception is unnecessary while taking non-enzyme-inducing antibiotics.

## Limitations

This review analyzes literature after 2000 to consider recent developments in interaction studies between antibiotics and COCs alongside contemporary clinical practices. While the prior studies form a foundational piece of literature, their exclusion could result in oversights regarding some prescribing practices that have lessened the interactions highlighted by the more recent research. Future studies should consider those perspectives that enhance the understanding of the interactions over time. It is important to mention that this review, focusing on the impact of antibiotics on COCs, does not discuss all the drugs that induce CYP enzymes or other hormones, such as some antiepileptic and antiretroviral drugs. Nonetheless, the studies included in this review represent existing literature on this topic.

## Conclusion

This analysis gives an in-depth review of the complex interaction between antibiotics and COCs. While most of the literature suggests that common antibiotics may not significantly impact COC efficacy, notable exceptions such as CYP3A4-inducing drugs such as rifampin should continue to be considered. Given the discrepancies, health care providers should remain vigilant about these potential interactions and adhere to guidelines such as those mentioned in this article while advising patients of possible interactions. Given the limited availability of high-quality clinical trials and randomized control studies, further research is necessary to ensure optimal patient outcomes.

## Abbreviations Used

ACOG: American College of Obstetricians and Gynecologists
CDC: Centers for Disease Control and Prevention
CHC: Combined Hormonal Contraception
COC: Combined Oral Contraceptive
CYP: Cytochrome P450
DCX: Dicloxacillin
DSG: Desogestrel
EM: Erythromycin
ENG: Etonogestrel
FSRH: Faculty of Sexual and Reproductive Healthcare
KTZ: Ketoconazole
LNG: Levonorgestrel
OCP: Oral Contraceptive Pill
POP: Progestin-Only Pill
RIF: Rifampicin
UPA: Ulipristal Acetate
USMEC: U.S. Medical Eligibility Criteria
WHO: World Health Organization
