# Supplementary material for: Exploring Antibiotic-Mediated Disruption of Enterohepatic Circulation and Combined Oral Contraceptive Efficacy: A Systematic Review
Source: Womens Health Rep (New Rochelle). 2025 May 19;6(1):599–604. doi: 10.1089/whr.2024.0199 (PMC12177323; doi:10.1089/whr.2024.0199)
Supplement: Supplementary Table S1 [file whr.2024.0199_supp_tables1.docx]

Supplemental Materials

Table 1 Search Strategy

| Database | Search String | Outcome |
| --- | --- | --- |
| PubMed | ("contraceptive devices"[MeSH Terms] OR "contraceptive agents"[MeSH Terms] OR "contraceptive pills"[Text Word] OR "hormonal contraception"[Text Word] OR "estrogen-progestin pills"[Text Word] OR "oral contraceptive pills"[Text Word] OR "birth control pill"[Text Word] OR "progestins"[MeSH Terms]) AND ("anti-bacterial agents"[MeSH Terms] OR "antibiotic therapy"[Text Word] OR "antimicrobial medication"[Text Word]) | 339 |
| Cochrane Library | Contraceptive OR “Contraceptive Agent” OR “oral contraceptive agent” OR “oral contraceptive” OR “hormonal contraceptive” OR “birth control pill” OR Progestins in Title Abstract Keyword AND antibiotics OR “antibiotic agent” OR anti-microbial in Title Abstract Keyword | 148 |
| Google Scholar | (Contraception OR contraceptive agents OR "contraceptive pills" OR "hormonal contraception" OR "estrogen-progestin pills" OR "oral contraceptive pills" OR "birth control pill" OR progestins) AND (antibacterial agents OR "antibiotic therapy" OR "antimicrobial medication") | 182 |
| Clinical Trial.gov | Antibiotics AND (“Oral contraceptives” OR “Birth control pills” OR “hormonal contraceptives”) | 43 |
| Total |  | 712 |
